# Quality assessment of yellowtail (*Seriola quinqueradiata*) meat cultured in an offshore floating flexible facility

**DOI:** 10.1002/fsn3.2898

**Published:** 2022-04-26

**Authors:** Masashi Ando, Wen Jye Mok, Yuji Maeda, Ryoji Miki, Takashi Fukuda, Yasuyuki Tsukamasa

**Affiliations:** ^1^ Department of Fisheries Faculty of Agriculture Kindai University Nara Japan; ^2^ 95438 Institute of Marine Biotechnology Universiti Malaysia Terengganu Terengganu Malaysia; ^3^ Nippon Steel Engineering Co., Ltd. Business Solution Center Fukuoka Japan

**Keywords:** coastal cage, meat quality, offshore floating flexible cage, yellowtail

## Abstract

Commercial aquaculture of yellowtail (*Seriola quinqueradiata*) is challenging, owing to deterioration of aquaculture environments. Offshore aquaculture may be a means of overcoming these problems. Here, we assessed the quality of flesh from offshore yellowtail (OY) bred for 1 year in an offshore floating flexible facility compared with coastal yellowtail (CY) cultured simultaneously in a coastal cage facility. The survival rate of the OY group was 94.46%, which was slightly lower than that of CY (98.18%). The feeding rate (feeding weight/fish weight) of CY was 0.4–0.5, whereas that of OY was only 0.3, possibly because poor weather conditions prevented feeding at the offshore facility. However, final fish weights did not differ significantly between both groups. In sensory tests, OY was inferior to CY in terms of oily taste. The lipid content in CY was significantly higher than that in OY. Hardness analysis revealed that OY muscles were harder than those of CY. There were no significant differences between OY and CY in overall sensory evaluations; thus, OY was judged as having equivalent value as a food product with CY. The redness of dark muscles was not significantly different on day 1 of refrigeration. However, the redness value of OY was significantly higher than that of CY on day 2. The inferior fattiness of OY relative to that of CY can be overcome by improving the feeding method. Therefore, offshore aquaculture with negligible environmental pollution may be effective for further development of aquaculture.

## Graphical abstract

Fish in the offshore floating flexible facility had lower fat contents and a harder texture. The redness of fish meat in the offshore floating flexible facility was maintained for a longer time. Offshore floating flexible culture can produce high‐quality yellowtail under eco‐friendly conditions.

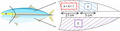

## 1 INTRODUCTION

Net cage fish culture effectively began with yellowtail (*Seriola quinqueradiata*) aquaculture (Harada, 1965), and fish have subsequently been cultured mainly in small‐scale net cages. Indeed, aquaculture has essentially been carried out in the same way in Japan since, and this method is being introduced all over the world. Fish culture facilities are commonly installed in the inner bays of coastal areas, where there are fewer waves or tides to interrupt feeding and landing. However, these installation conditions often cause organic matter, such as residual food and fish excrement, to accumulate directly under the fish culture net cages without being carried away. Various microorganisms inhabit the ocean floor, and some organic matter is mineralized through microbe purification. However, if the organic matter deposited exceeds the purification capacity, then the amount of nonmineralized organic matter increases, and hydrogen sulfide is produced from the organic matter via anaerobic decomposition, resulting a deteriorated aquaculture environment (Tsutsumi et al., 1991).

To overcome the problems associated with coastal fish culture nets, net facilities can be installed offshore. In tidal sea areas, organic matter is unlikely to accumulate on the sea floor, and the hypoxic seawater near the sea floor is replaced from above via vertical mixing. Thus, the water quality is unlikely to deteriorate (Keiyu & Yokota, 2012). However, the waves are larger in the open sea, particularly when typhoons occur, and the risk of damage to fish culture facilities is higher. In response to rough seas, a floating flexible cage design was developed (Kimura, 1976). Nevertheless, this approach involves offshore facilities, meaning that workers must move offshore for feeding and landing the cultured fish. Furthermore, it is difficult to approach the facility on days when the sea is rough. Therefore, as a countermeasure, remote‐controlled feeding has been developed in recent years.

The meat quality of cultured fish is often judged to be better than wild‐caught fish, especially those caught by trawling due to the handling, stunning, and slaughtering methods employed (Borderías & Sánchez‐Alonso, 2011). Nevertheless, it remains uncertain whether there are differences between the fish meat quality from different culturing systems. Offshore floating flexible fish culture facilities have introduced various improvements, but the quality of fish from this cultured system have not been compared with other culture systems. Therefore, in the current study, we evaluated the meat quality of yellowtail cultured in the floating flexible cage and that of yellowtail cultured in a coastal culture facility. We aimed to clarify the difference between the meat quality of yellowtail cultured in offshore and coastal cages.

## 2 MATERIALS AND METHODS

### 2.1 Aquaculture facilities and breeding conditions

**FIGURE 1:**
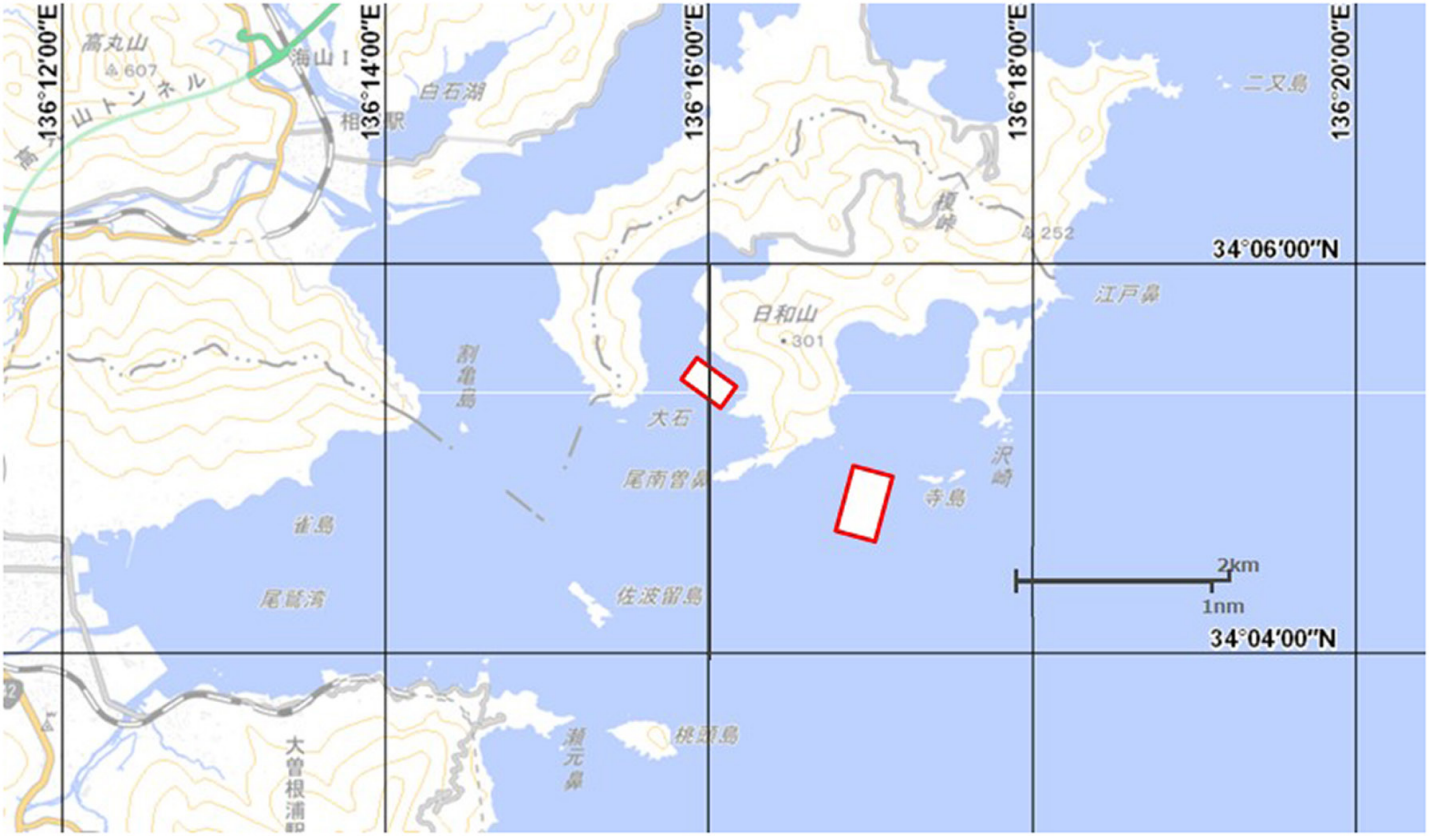
Site map of farming facilities. Adapted from MDA Situational Indication Linkages (2019; https://www.msil.go.jp/; accessed on September 20, 2019, with modifications)

We used one offshore farming fish culture facility and one coastal farming fish culture facility. Details of the locations of the facilities are presented in Figure 1 (MDA, 2019) and Table 1. Table 2 shows the culturing conditions and data pertaining to the cultured individuals. In addition, a tidal current meter (INFINITY‐EM; JFE Advantech Co., Ltd.) was installed at each facility to measure the tidal velocity in each area. The test fish were introduced into the offshore net cage in April 2017. Satiety feeding was performed once daily.

**TABLE 1:** Details of farming facilities and their installation conditions

	Offshore	Coastal
Size of net (m)	30 × 30 × 20	10 × 10 × 10
Material of net	Chemical fiber	Metal (bottom) and chemical fiber (sides)
Floating condition	Float and sink	Floating
Depth of setting site (m)	64	30
Average streaming rate at the setting site (knot)	0.3	0.2

**TABLE 2:** Yellowtail culturing conditions

	Offshore	Coastal
Culturing period	April 2017 to March 2018	April 2017 to March 2018
Number of individuals at the start	32,372	4279
Food	Formula feed, IK16	Formula feed, IK16
Feeding methods	Blowing to a depth of 1 m, feeding to satiety	Whisper Feeder from above, feeding to satiety
Feeding intervals (days)	1–2	1–2
Maximum feeding time (min)	100	20
Maximum culturing density (kg/m^3^)	10	20
Body weight at the start (kg, average ±SE)	1.90 ± 0.21	1.67 ± 0.22
Body weight at the end (kg, average ±SE; *n* = 6)	5.13 ± 0.21	5.78 ± 0.25
Survival rate (%)	94.46	98.18

### 2.2 Sample collection

**FIGURE 2:**
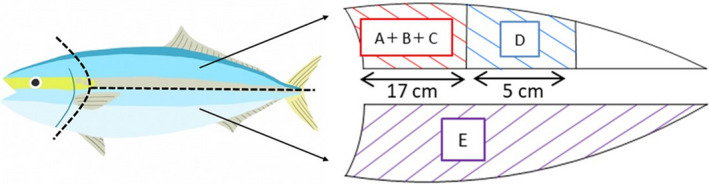
Muscle parts of yellowtail used for each analytical item. A: Sensory test; B: firmness; C: K value; D: dark muscle color; E: proximate composition, free amino acids, fatty acids, ascorbic acid, α‐tocopherol, and iron

All experiments and protocols were performed in strict accordance with the Guiding Principles for the Care and Use of Research Animals adopted by the Kindai University Committee on Animal Research and Bioethics. On March 14, 2018, six live yellowtail individuals cultured in offshore (OY) or coastal (CY) facilities were picked and used as samples for various analyses. Different parts of the samples were used for various analyses, as shown in Figure 2. Part A was used for sensory tests; part B was used to measure hardness; part C was used to measure the K value; part D was used to measure dark muscle color; and part E was used to assess the proximate composition, free amino acid, fatty acid, vitamin C, vitamin E, and iron contents. Body weight was measured during culturing from images obtained using a camera in net cages (AM100; AQ1 Systems Co.). The body length and height were measured automatically by the AM100 analyzer from images captured using a camera, and the data were input into built‐in software. A formula to calculate yellowtail weight is included in the software.

### 2.3 Sensory test

Part A (Figure 2) was cut out, vacuum sealed, and packed into ice‐filled foam containers for delivery. A sensory test was conducted on March 15, 2018, 1 days after landing. The muscle was cut to a thickness of 1 cm perpendicular to the body axis and used as‐is for the sensory test. The sensory test was conducted by 24 well‐trained inspectors (three men and 21 women). The evaluated parameters were as follows: dark muscle color, ordinary muscle color, acidity, fresh odor, oily taste, hardness, texture, and comprehensive features. For all parameters, coastal aquaculture samples were scored as 0 and evaluated. The parameters were scored as follows over seven stages: dark muscle color, vivid (+3 points) to not vivid (−3 points); ordinary muscle color, strong white (+3 points) to strong red (−3 points); sour and raw odor, strong (+3 points) to weak (−3 points); oily taste, oily (+3 points) to nonoily (−3 points); hardness, hard (+3 points) to soft (−3 points); tongue feel, smooth (+3 points) to not smooth (−3 points); and comprehensive evaluation, good (+3 points) to bad (3 points) (Muñoz et al. 1992).

### 2.4 Hardness

Part B (Figure 2) was cut out, vacuum sealed, placed in an ice‐packed foam container, and transferred from the processing site to the laboratory. The hardness of samples was measured on March 15, 2018, 1 d after landing. The muscle samples were cut into 1.5‐cm‐thick pieces perpendicular to the body axis. A 5‐mm‐diameter cylindrical plunger was attached to a creep meter (RE‐3305; Yamaden), and the sample stage ascending speed was set to 5 mm/s. The plunger entered the body in the same direction as the body axis, and the load when the specimen disintegrated was defined as the hardness.

### 2.5 K value

Part C (Figure 2) was cut out, vacuum sealed, placed in an ice‐packed foam container, and transferred from the processing site to the laboratory. After freezing at −20°C on March 15, 2018, 1 day after landing, the samples were thawed when necessary and homogenized by adding 5 ml of 10% trichloroacetic acid to 3 g of the sample. Using the filtrate obtained by filtering the homogenate through filter paper (5A; Advantec, retained particle size: 7 μm), the K value was measured using a freshness measuring instrument (KV‐202; Central Science Co., Ltd., Japan).

### 2.6 Proximate composition

The proximate composition was measured using part E (Figure 2). Moisture content was determined by drying the samples at 105°C under atmospheric pressure. Ash content was determined by heating the sample at 550°C for 6 hr. Crude protein content was analyzed using the Kjeldahl method (Kjeldahl, 1883). Crude fat content was determined using the Soxhlet extraction method (Soxhlet, 1879). Total carbohydrate content was calculated by subtracting the percentages of fat, protein, ash, and moisture content from 100.

### 2.7 Free amino acids

After adding 25 ml of 10% sulfosalicylic acid solution to 6 g of the sample collected from part E (Figure 2) and extracting via shaking, the pH was adjusted to 2.2 with 3 M sodium hydroxide and the volume was adjusted to 50 ml. The content of free amino acids in the extract was measured using an amino acid analyzer (L‐8900; Hitachi).

### 2.8 Fatty acid composition

Lipids were extracted from the sample (50–100 g) collected from site E (Figure 2) by chloroform/methanol extraction (MEXT, 2016). The extracted lipid was saponified by adding 0.5 M sodium hydroxide‐methanol solution and then methylesterified by adding a boron trifluoride methanol complex methanol solution. Extraction was performed with hexane and saturated saline. The hexane layer was injected into a gas chromatograph (7890B; Agilent Technologies) equipped with a capillary column (DB‐23; Agilent Technologies) and an FID, and the content of free fatty acids was measured.

### 2.9 Evaluation of dark muscle color

A fillet (Figure 2D) was placed in a plastic container, which was immersed in ice water during transport. One day after landing, the color difference was measured using a color difference meter (ZE 6000; Nippon Denshoku Industries Co., Ltd.), and the sample was returned to its container. The sample was stored at 4°C for another day, and the color difference was measured again.

### 2.10 Iron

A sample (5–20 g) collected from part E (Figure 2) was burned to ash at 500°C for over 5 hr, extracted with 20% hydrochloric acid, filtered, and the volume adjusted to 50 ml with distilled water. Iron content was measured by inductively coupled plasma atomic emission spectroscopy (735‐ES; Agilent Technologies; MEXT, 2016).

### 2.11 Total ascorbic acid content

A sample (2–6 g) collected from part E (Figure 2) was used to determine the total ascorbic acid content (MEXT, 2016). The sample was homogenized with 5% metaphosphoric acid. After making the volume up to 50 ml, the sample was centrifuged (450 × *g*, 5 min) and filtered through filter paper (no. 3; Advantec; retained particle size: 5 μm). Osazone was formed by adding 0.2% 2,6‐dichlorophenolindophenol solution to the filtrate. After adding ethyl acetate, the solution was shaken, and the ethyl acetate layer was analyzed using a high‐performance liquid chromatograph (LC‐20AT; Shimadzu), with the following parameters: column, SenshuPAK Silica‐1100‐*N* (Senshu Chemical); φ4.6 mm × 10 cm; column temperature, 40°C; mobile phase, ethyl acetate:n‐hexane:acetic acid:water = 60:40:5:0.5 (v/v/v/v); flow rate, 1.5 ml/min; detection wavelength, 495 nm.

### 2.12 α‐Tocopherol

About 0.5–3 g samples were collected from part E (Figure 2), 2 ml of 1% NaCl (w/v), 0.3 g pyrogallol, 10 ml ethanol, and 60% potassium hydroxide (w/v) were added, and the mixture was then heated at 70°C for 30 min (MEXT, 2016). After cooling, 1% NaCl (w/v) and hexane‐2‐propanol‐ethyl acetate (9:1.5:1, v/v/v) were added to the solution, and extraction was performed with shaking. After centrifugation at 450 × *g* for 5 min, the organic solvent layer was evaporated. After dissolving in hexane, the content of *α*‐tocopherol was measured using a high‐performance liquid chromatograph with a fluorescence detector. The following parameters were used: column, YMC‐PAK SIL‐06 S‐5 (YMC Co., Kyoto, Japan); φ4.6 mm × 25 cm; column temperature, 40°C; mobile phase, n‐hexane:2‐propanol:acetic acid = 1000:6:5 (v/v/v); flow rate, 1.5 ml/min; excitation wavelength, 298 nm; and detection wavelength, 325 nm.

### 2.13 Statistical analysis

Significant differences between the measured values for OY and CY were analyzed using Student's *t*‐test, with *p* <.05 being set as the cut‐off for statistical significance. Except for sensory tests, differences in test results between the average OY and CY values were tested. In the sensory test, the results of CY meat were set to 0, and OY was evaluated relative to those results. Finally, the differences between the average values of the two samples were analyzed. The statistical analyses were performed in Excel (Microsoft Co.).

## 3 RESULTS AND DISCUSSION

### 3.1 Changes in food consumption and fish weight

**FIGURE 3:**
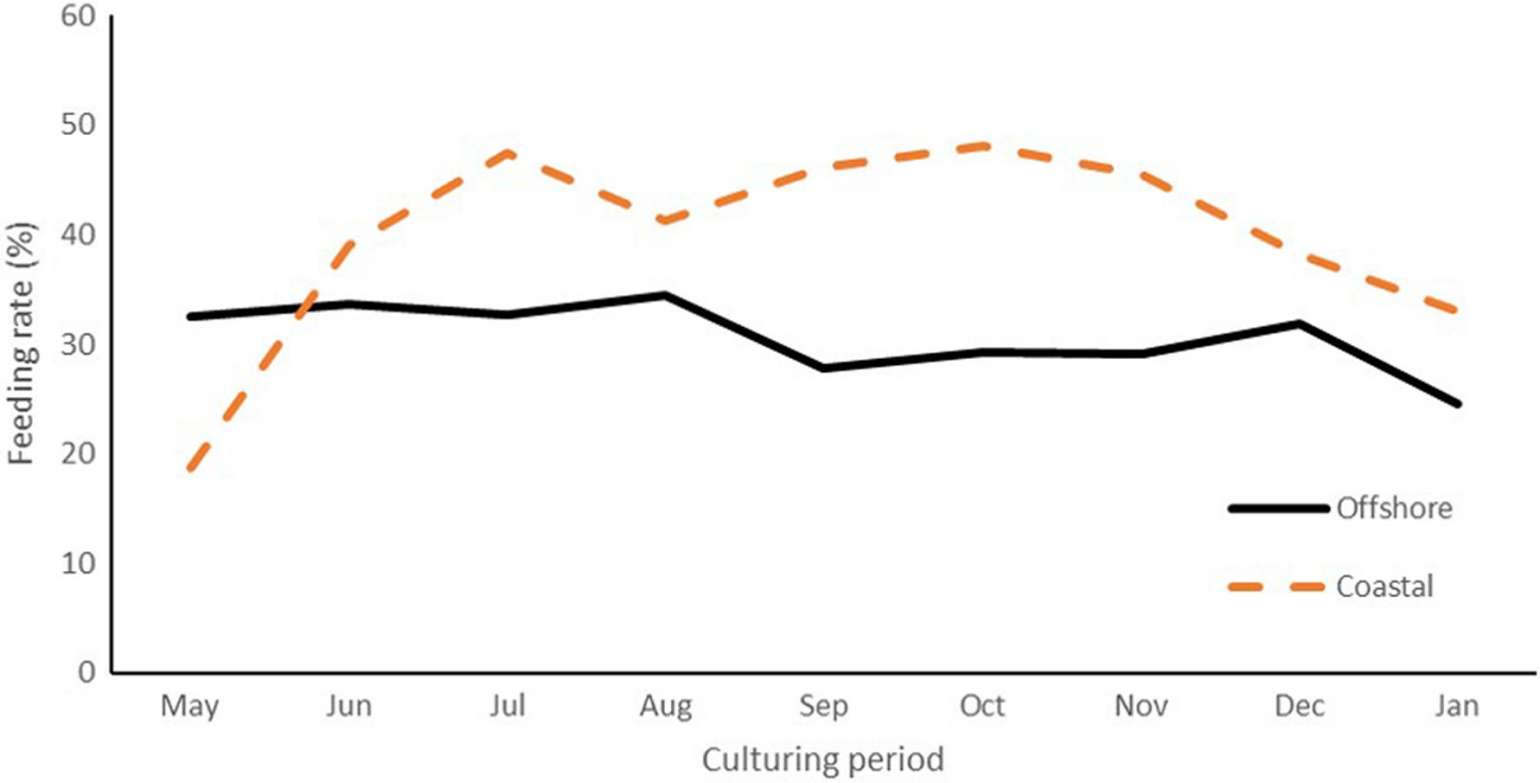
Feeding rate during the culturing period. The values were calculated as follows: feeding rate = 100 x total amount of feed given per month / total fish weight at each farming facility per month. Total fish weight was calculated from the fish number, and each fish body was measured from video images

Figure 3 shows changes in food intake during the culturing period. Food consumption relative to body weight was higher in CY than in OY, except during the early period of culturing. The estimated body weights of OY and CY at the start of culturing were 1.90 ± 0.21 and 1.67 ± 0.22 kg, respectively, and those at the end of culturing were 4.94 ± 0.61 and 5.64 ± 0.63 kg, respectively; however, these values were not significantly different (Table 2). The survival rates were 94.5% for OY and 98.18% for CY (Table 2).

### 3.2 K value, sensory test, hardness, and proximate composition

When the K value, a freshness index, was analyzed, no significant differences were found on day 1 after sampling (Table 3). Therefore, OY is likely equivalent to CY in terms of freshness deterioration rate. This indicated that the landing methods for both culture systems did not cause freshness deterioration, despite OY being further from land and taking a longer time to transport.

**TABLE 3:** Physicochemical properties of yellowtail flesh at the end of culturing in offshore and coastal aquaculture facilities (average ±SE, *n* = 6)

	Offshore	Coastal
K value (%, 1 day after chilled storage)	5.2 ± 0.4	5.0 ± 0.6
Firmness (*N*, 1 day after chilled storage)	1.82 ± 0.03*	1.71 ± 0.03
Moisture (g/100 g)	62.7 ± 0.6*	60.2 ± 0.7
Protein (g/100 g)	23.5 ± 0.1*	22.4 ± 0.2
Lipid (g/100 g)	13.0 ± 0.6*	16.5 ± 0.8
Ash (g/100 g)	1.10 ± 0.00	1.07 ± 0.02
Carbohydrate (g/100 g)	0.03 ± 0.03	0.07 ± 0.05
Energy (kcal/100 g)	211.2 ± 5.3*	238.2 ± 7.1

*Statistical differences were analyzed using Student's *t*‐tests (*p* <.05).

In sensory tests, when the value of CY was set as 0, OY tasted significantly less oily (−0.42 points), and the hardness was significantly higher (0.46 points; Table 4). When equivalent CY and OY samples were subjected to physicochemical analysis, OY exhibited a significantly lower lipid content, and the hardness was significantly higher than CY (Table 3). The sensory test results of both CY and OY were consistent with the results of the physicochemical analysis.

**TABLE 4:** Evaluation of yellowtail meat quality using sensory tests in individuals reared in an offshore sink‐float type farming facility compared with those cultured in a coastal facility (average ±SE, *n* = 4)

Color of dark muscle	−0.292 ± 0.172
Color of ordinary muscle	0.167 ± 0.281
Sour taste	−0.250 ± 0.198
Raw smell	0.292 ± 0.181
Oiliness	−0.417 ± 0.186*
Firmness	0.458 ± 0.195*
Tongue feel	−0.250 ± 0.135
Overall evaluation	−0.292 ± 0.224

*Statistical differences were analyzed using Student's *t*‐tests (*p* <.05).

In general, the anatomical composition of fish is strongly influenced by environmental factors and diet is considered the chief contributor (Orban et al., 2007). The oily taste was inferior in OY owing to feeding challenges. From September to November, there were days when the fish could not be fed due to storms caused by typhoons, and there were significant differences in the amount of feed given for the body weight during that period (Figure 3). Moreover, the offshore facility had a large capacity (30,000 fish), and it took 17 d to land the fish during the landing period due to a storm. During this period, the fish were not fed, and the analyzed fish were landed on the last day of the 17‐d landing period. Therefore, the individuals analyzed at this time had fasted for 17 d.

Watanabe et al. (2000) showed that the muscle fat of yellowtails (with an initial average body weight of 506 g) was reduced from 16.2% to 15.2% during 20 days of fasting in a 3 × 3 × 3 m cage due to the narrow coastal facility preventing swimming. In this study, the lipid content of OY however was further reduced by fasting in the offshore facility that had strong currents. This differences in lipid content could be due to higher swimming activities at the offshore facilities that had stronger currents. In principle, coastal culture facilities are installed in locations where there is almost no tidal current because such a current could cause the facility to become unstable, resulting in net cages becoming deformed, causing fish to rub and experience stress, consequently leading to injury and illness. In contrast, the offshore facility used in the current study was installed at a location with a tidal current of approximately 0.3 knots (~0.55 km/h); the tidal current at the coastal facility was approximately 0.2 knots. Thus, the tidal current was not considerably fast in either of the sea areas (Table 1). However, because the offshore facility was constantly subjected to these environmental conditions, the water movement was considerably higher than that of the coastal facility. Therefore, this may have resulted in increased energy consumption, which may have contributed to the low lipid content of the OY fish (Table 5).

The feeding time for CY was approximately 20 min per feeding, whereas that of OY was approximately 100 min due to the larger cage size and greater number of individuals in the cage. The cage size and the distribution of the feed in the cage may have caused the OY to become more excited and swim more vigorously than the CY. A previous study demonstrated that plasma‐free fatty acid levels decrease due to greater utilization of free fatty acids by muscles when swimming intensity increases (Li et al., 2015). Thus, daily changes in the exercise state increased the energy consumption in OY more than in CY, reducing the lipid contents in the muscles of OY specimens.

Next, we investigated why the muscles of OY were significantly harder. The skeletal muscles consist of muscle fibers and connective tissue (Lieber, 2002). As the muscle consists of approximately 90% muscle fibers and 10% of connective and fat tissues, depending on species, the muscle fibers are the main component influencing the hardness evaluation (Listrat et al., 2016). Listrat et al. (2016) also stated that muscle fiber composition is also influenced by breed, gender, age, physical activity, environmental temperature, and feeding practices. With OY experiencing a greater exercise load than CY due to the ocean currents in the facility as well as the feeding practices, the muscle fibers that contribute to stiffness were relatively well developed due to either hyperplastic (increase in fiber number) or hypertrophic (increase in fiber size) growth (Kiessling et al., 2006). This hypothesis was supported by significant increases observed in muscle proteins in OY (Table 3). Tachibana et al. (1988) reported that forcing cultured red sea bream into an artificially created water stream improved the physical strength of the myofibrils to the same level as that of wild red sea bream. As in that report, OY were placed in an environment that led to greater exercise intensity than the coastal environment for approximately one year, which strengthened the myofibrils and increased the hardness of the muscles.

Another factor that affects muscle stiffness is the connective tissue (Lieber, 2002) due to its composition and structure (Astruc, 2014), though it only accounts for a small percentage of the muscle overall. The connective tissue connects the muscle fibers, and its influence on the physical properties of muscles cannot be ignored. In fish, muscles tend to soften over time after death, and one cause is the weakening of connective tissue (Ando et al., 1993). In general, the connective tissue is divided into three structures, namely the epimysium (surrounds whole skeletal muscle), perimysium (separates bundles of muscle fibers within the muscle), and endomysium (surrounds each muscle fiber) (Astruc, 2014). Of these three structures, perimysium is most variable in terms of composition, and it can change with nutrition and exercise (Astruc, 2014). Therefore, if the connective tissue of the OY muscles is strengthened through excessive swimming activities by thickening the perimysium layer, then the muscles would be less likely to soften and would consequently be harder than the CY muscles.

In fish muscles, the total moisture and lipid contents are nearly constant (Saeki & Kumagai, 1979). Consistent with this, the muscles of OY had greater moisture content (Table 3) but less lipid content than those of CY. The amount of energy in OY was also significantly lower, owing to the effects of lower lipid content (Table 3). Therefore, our results indicated that OY muscles had lower calorie content and higher protein content values than CY muscles. Without temporary fasting, as described above, OY may have the same lipid content as CY; however, OY had higher protein content and was superior to CY in terms of nutrition, indicating its potential as a new aquatic food source.

### 3.3 Dark muscle color

**FIGURE 4:**
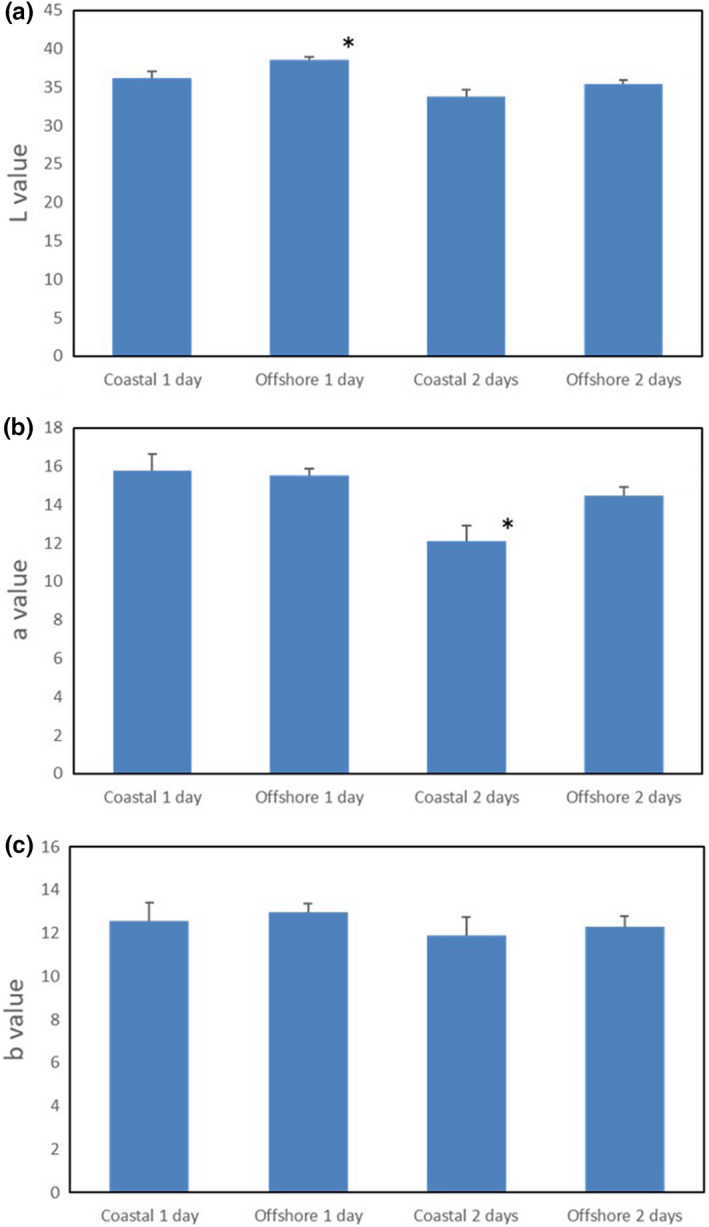
Change in dark muscle color of yellowtail during chilled storage. A: L value, B: a* value, C: b* value. *Statistical differences between the coastal and offshore individuals on the same storage day were analyzed using Student’s t‐test (*p* < .05). *n* = 6

There were no significant differences in the color of dark muscles based on sensory tests (Table 4). However, there were significant differences in L and a* values (Figure 4). With respect to the L value, which indicates brightness, OY showed slightly higher values than CY 1 d after landing (Figure 4A). However, this difference was not observed after 2 d of storage (Figure 4A). In addition, the a* value of CY was significantly lower after 2 d of storage than OY (Figure 4B). Because the positive a* value represents redness intensity, this result indicated that the dark muscles of CY were significantly less red than those of OY after 2 d of storage. Conversely, there were no significant differences in b* values (Figure 4C).

The degree of redness in fish meat greatly affects fish assessment (Clydesdale, 1991). The red color is derived from myoglobin, the sarcoplasmic heme protein primarily responsible for the meat color (Suman & Joseph, 2013). Myoglobin is bright red when oxygenated; however, decreased oxygen levels reduce the redness as it is progressively converted to metmyoglobin (“yake”) after death (Bito, 1964). “Yake” is a phenomenon that is considered by farmers to severely deteriorate tuna and yellowtail meat. A 2‐days period after landing, which must also include the period of purchase and consumption by consumers, results in significant differences in the redness of meat in the current study. Therefore, the commercial value of OY, which can maintain its redness for over a longer period, may be higher than that of CY.

Discoloration of meat occurs when the iron (II) contained in myoglobin is oxidized to iron (III) (Bito, 1964). There are several factors that promote discoloration, including increased body temperature owing to increased exercise activity during landing and low muscle pH (Konagaya, 1982). However, in the current study, the picking and cooling methods were exactly the same; therefore, the discoloration must have been related to other factors. Lipid oxidation can also promote myoglobin oxidation (Sohn & Ohshima, 2010; Wang et al., 2021). Unsaturated fatty acids located near the cellular membranes of myofiber in fish meat can easily be oxidized; therefore, muscles rich in lipids are relatively easily oxidized (Wang et al., 2021). Thus, as oxidation of lipid and myoglobin happens concurrently, the meat is easily discolored. Wang et al. (2021) also stated that although polyunsaturated fatty acids (PUFAs) do not directly cause discoloration, the relationship between PUFAs might be mediated through the interactions of heme iron and vitamin E that can cause discoloration of meat through lipid and myoglobin oxidation. In the current study, OY had a significantly lower lipid content (Table 3) in the ordinary muscle and lower still in the dark muscle, while the PUFA content was also lower (Table 5) than CY. Therefore, lipid oxidation proceeded slower in the dark muscle of OY owing to the significantly lower lipid content and myoglobin oxidation, suggesting that discoloration did not readily occur.

**TABLE 5:** Free amino acid contents in ordinary muscle of yellowtail from offshore and coastal aquaculture facilities (mg/100 g, average ±SE, *n* = 6)

	Offshore	Coastal
Arginine	3.17 ± 0.31	4.67 ± 0.88
Lysine	31.67 ± 4.20	30.17 ± 2.87
Histidine	1121.67 ± 30.49	1086.67 ± 21.55
Phenylalanine	1.67 ± 0.21	1.67 ± 0.21
Tyrosine	2.33 ± 0.21	3.17 ± 0.31
Leucine	4.17 ± 0.31	3.83 ± 0.60
Isoleucine	3.00 ± 0.26*	1.50 ± 0.29
Methionine	1.83 ± 0.17*	1.00 ± 0.00
Valine	4.00 ± 0.37*	2.50 ± 0.22
Alanine	20.17 ± 1.01*	27.00 ± 1.88
Glycine	7.00 ± 0.58*	4.83 ± 0.60
Proline	1.40 ± 0.24*	17.00 ± 5.11
Glutamic acid	20.67 ± 2.09*	8.67 ± 1.96
Serine	4.50 ± 0.34	3.83 ± 0.91
Threonine	4.67 ± 0.67	3.67 ± 0.67
Glutamine	14.50 ± 1.63*	4.67 ± 1.71
Ornithine	1.50 ± 0.34*	4.83 ± 1.14
Taurine	0.025 ± 0.002*	0.03 ± 0.002
Anserine	0.083 ± 0.003	0.09 ± 0.004

*Statistical differences were analyzed using Student's *t*‐tests (*p* <.05).

### 3.4 Free amino acids

Table 5 shows the free amino acids in ordinary muscle. There were 10 amino acids that were significantly different between OY and CY. Among them, four amino acids, namely, proline, glutamic acid, glutamine, and ornithine, significantly differed in terms of the quantity ratio (OY/CY). Considerably higher values were observed for glutamic acid (238%) and glutamine (311%). Glutamic acid is a typical substance that confers an umami flavor; therefore, it is an important substance for determining food quality (Yamaguchi & Ninomiya, 2000). Although there were no parameters related to taste in the sensory test performed in the current study, there may be differences in the taste of the muscle.

**TABLE 6:** Fatty acid compositions in ordinary muscle of yellowtail from offshore and coastal aquaculture facilities (g/100 g, average ±SE, *n* = 6)

	Offshore	Coastal
Total fatty acids	10.32 ± 0.27*	13.33 ± 0.74
SFA	2.83 ± 0.07*	3.57 ± 0.19
MFA	3.80 ± 0.10*	5.05 ± 0.29
PUFA	3.68 ± 0.10*	4.72 ± 0.27
*n*−3	2.62 ± 0.07*	3.23 ± 0.18
*n*−6	1.07 ± 0.03*	1.48 ± 0.10
*n*−3/*n*−6	2.46 ± 0.06*	2.19 ± 0.04
*n*−9	2.87 ± 0.07*	3.88 ± 0.22
C14:0	0.483 ± 0.017*	0.567 ± 0.021
C16:0	1.933 ± 0.049*	2.450 ± 0.126
C16:1	0.583 ± 0.017*	0.733 ± 0.033
C18:0	0.417 ± 0.017*	0.533 ± 0.033
C18:1n−9	2.650 ± 0.062*	3.617 ± 0.206
C18:1n−7	0.350 ± 0.022	0.433 ± 0.033
C18:2n−6	1.050 ± 0.022*	1.400 ± 0.086
C18:3n−3	0.200 ± 0.000*	0.267 ± 0.021
C18:4n−3	0.200 ± 0.000	0.200 ± 0.000
C20:1n−9	0.217 ± 0.017	0.267 ± 0.021
C20:4n−6	ND	0.100 ± 0.000
C20:4n−3	ND	0.100 ± 0.000
C20:5n−3	0.717 ± 0.031*	0.883 ± 0.048
C22:5n−3	0.283 ± 0.017*	0.350 ± 0.022
C22:6n−3	1.217 ± 0.031*	1.467 ± 0.071

*Statistical differences were analyzed using Student's *t*‐tests (*p* <.05).

Glutamine content was higher in OY than in CY. Glutamine is an excitatory neurotransmitter that affects brain activity (Gasic & Hollmann, 1992). Additionally, glutamine has been reported to improve intestinal immunity (Ueno & Tanizawa, 2006), and increased glutamine has been reported to reduce the incidence of infection (Novak et al., 2002). Although these studies were conducted in humans, similar results are expected to occur in fish after exercise, and it may be possible to produce disease‐resistant cultured fish. Studies on the relationships between exercise and glutamine content have shown that muscle breakdown and synthesis processes become more active with exercise, necessitating higher glutamine content (Rennie et al., 1981). This can explain the higher glutamine content observed in OY.

Glutamic acid and glutamine content in fish are important for maintaining the health of humans who consume fish. Supplementing dietary glutamine may also improve fish quality by inhibiting glycolysis and enhancing antioxidative functions in skeletal muscle as well as its water‐holding capacity; thus, it may improve the color and firmness of their meat (Li et al., 2020). The current study may support these findings.

Proline (8%) and ornithine (31%) were less abundant in OY than in CY. Proline is synthesized from ornithine (Delauney & Verma, 1993). Proline is one of the major amino acids contained in the collagen of fish muscle (Li & Wu, 2018); thus, the higher proline content may indicate higher collagen content that may cause the muscles to soften after during short‐term chilled storage (Sato et al., 1997). In particular, proline content is thought to be depleted by exercise (Williams et al., 1950), which may explain the results of the current study.

These differences in the concentrations of free amino acids were observed despite fish being fed the same diet. Thus, these differences could be ascribed to differing exercise levels, leading to differences in amino acid synthesis and metabolism. If proline and ornithine were depleted through exercise, then their loss could be mitigated by rearing the fish using a mixed feed supplemented with these amino acids.

### 3.5 Fatty acid composition

Fatty acids are essential for life due to their roles as sources to produce membrane constituents, energy, and metabolic and signaling mediators (Pereira et al., 2013). Fatty acid concentrations were significantly higher in CY than in OY (Table 6). PUFAs were more abundant than saturated fatty acids and less abundant than monounsaturated fatty acids in both CY and OY, but there were no significant differences in the quantity ratio of each fatty acid. Among the fatty acid components, some PUFAs, such as eicosapentaenoic acid (EPA, C20:5n‐3) and docosahexaenoic acid (DHA, C22:6n‐3), are essential fatty acids that cannot be synthesized by humans but play important roles in the prevention of many diseases, making them important determinants of food quality (Swanson et al., 2012; Zhang et al., 2020). The fatty acid concentration in the muscle is affected by various factors, such as feed, species, body weight, age, sex, and season (Wood et al., 2008). In the current study, their concentrations may be controllable by feeding, which leads to the differences in weight and it may be possible to shift OY to a preferable fatty acid composition by stabilizing the amount of feeding.

### 3.6 Iron, ascorbic acid, and α‐tocopherol

The iron concentration in OY showed a small but significant decrease compared with that in CY (Table 7). The slower progress of discoloration of OY meat (Figure 4) may be related to the low iron (ion) concentration. Because iron ions catalyze the oxidation reaction (Tichivangana & Morrissey, 1985), it is possible to enhance the oxidation reaction from Fe^2+^ to Fe^3+^ in myoglobin. Although the quantitative iron value does not distinguish between metals or ions, there may be differences in the concentrations of iron ions between the two fish groups.

**TABLE 7:** Iron, ascorbic acid, and α‐tocopherol content in ordinary muscle of yellowtail from offshore and coastal aquaculture facilities (mg/100 g, average ±SE, *n* = 6)

	Offshore	Coastal
Iron	0.25 ± 0.01*	0.28 ± 0.01
Ascorbic acid	10.8 ± 1.0	13.5 ± 1.4
*a*‐Tocopherol	3.93 ± 0.22	4.37 ± 0.25

*Statistical differences were analyzed using Student's *t*‐tests (*p* <.05).

The ascorbic acid and α‐tocopherol contents did not differ significantly between OY and CY (Table 7). Although these components can be derived from food, differences in the amount of feed consumed by the fish did not affect their levels.

### 3.7 Future challenges for offshore fish culture

In overall sensory test evaluations, the meat quality of OY was equivalent to that of CY (Table 4). The inferior oily taste of OY was mainly caused by insufficient feeding owing to poor weather conditions. Therefore, if continuous feeding even under poor weather conditions can be achieved by improving automatic feeding machines, then yellowtail with properly developed muscles and a favorable oily taste can be produced.

In recent years, the importance of sustainable production has increased. Offshore aquaculture facilities can be used to culture fish with equivalent or better quality than previous production techniques, while also reducing environmental pollution; thus, this approach could be an important means of culturing fish. However, major issues remain, such as construction and maintenance costs, which must be overcome to preserve the sea environment.

## 4 CONCLUSION

OY and CY specimens were simultaneously cultured in a float‐sink offshore fish culture facility and a coastal facility and their meat quality was compared. OY was inferior to CY in terms of oily taste; however, the meat hardness and dark muscle color retention time values of OY exceeded those of CY, and the overall evaluation was equivalent. Improved feeding methods could sufficiently overcome the inferior fattiness of OY. Therefore, offshore aquaculture offers an effective means of developing a sustainable aquaculture environment with less environmental pollution.

## CONFLICT OF INTEREST

The authors declare that they have no known competing financial interests or personal relationships that could have influenced the work reported in this paper.

## AUTHOR CONTRIBUTIONS

**Masashi Ando:** Conceptualization (lead); Investigation (lead); Supervision (lead); Validation (lead); Visualization (equal); Writing – original draft (lead); Writing – review & editing (equal). **Wen Jye Mok:** Validation (supporting); Visualization (supporting); Writing – original draft (supporting); Writing – review & editing (lead). **Yuuji Maeda:** Data curation (equal); Methodology (equal); Project administration (equal); Resources (equal). **Ryoji Miki:** Data curation (equal); Methodology (equal); Project administration (equal); Resources (equal). **Takashi Fukuda:** Data curation (equal); Software (equal); Supervision (equal). **Yasuyuki Tsukamasa:** Data curation (equal); Software (equal); Supervision (equal).

## ETHICAL APPROVAL

All experiments and protocols were performed in strict accordance with the Guiding Principles for the Care and Use of Research Animals adopted by the Kindai University Committee on Animal Research and Bioethics.

## Data Availability

Data sharing not applicable – no new data generated, or the article describes entirely theoretical research.
